# Supplementary material for: GT61 β‐1,2‐xylosyltransferases define a conserved xylan modification in gymnosperm and Arabidopsis primary cell walls
Source: Plant J. 2025 Nov 2;124(3):e70545. doi: 10.1111/tpj.70545 (PMC12579940; doi:10.1111/tpj.70545)
Supplement: Supplementary file 3 — Figure S1. Difference in xylan structure in needles (N) and wood (W). Figure S2. Structural analysis of Y xylo‐oligosaccharide from Metasequoia needles. Figure S3. Differential activity of GT61 xylosyltransferases from Pseudotsuga menziesii group II (PmXYXT2) and III (PmXYXT1). Figure S4. Subcellular localisation of PmXYXT2 in Nicotiana benthamiana leaves. Figure S5. PACE analysis of xylan oligosaccharides generated by ectopic expression of GT61 xylosyltransferases in Arabidopsis stem. Figure S6. Arabidopsis AtXYXT2 and AtXYXT3 mutants. Figure S7. Analysis of seed phenotype in atxyxt1 atxyxt2 atxyxt3 mutants. Figure S8. Analysis of macro‐phenotype of atxyxt1 atxyxt2 atxyxt3 triple mutants. [file TPJ-124-0-s001.zip › Supporting Figure legends.docx]

**Supporting Figure** **S1 Difference in xylan structure in needles (N) and wood (W)**PACE analysis of AIR from Pinus radiata, Pinus abies, *Pseudotsuga menziesii* and *Metasequoia glyptostroboides* hydrolysed with xylanase GH11. Note the difference in ratio of unknown oligosaccharide A and Y. S: Standard of xylo-oligosaccharides X_1_-X_6_.

**Supporting Figure** **S2** Structural analysis of Y xylo-oligosaccharide from Metasequoia needles
**A** 2D *1*H,*13*C-HSQC spectrum of a metasequoia xylan oligosaccharide obtained at 800 MHz in D*_2_*O at 298 K. Letters a–h correspond to the residues labelled in the Symbol Nomneclature For Glycans (SNFG) structure shown in the insert, bottom right, while i corresponds to a further β-D-xylose residue and j–l to arabinose residues, where there was no linkage evidence for any of these additional residues to the structure identified. α and β indicate the reducing end anomer conformation that the corresponding peak relates to.
**B** NMR analysis of the T-β-D-Xyl-(1 → 4)-β-D-Xyl-(1 → 4)-β-D-Xyl-(1 → 4)-β-D-Xyl-(1 → 4)-β-D-Xyl-(1 → 4)-β-D-Xyl oligosaccharide backbone structure, using *1*H strip plots for *1*H,*1*H-TOCSY (black) and *1*H,*1*H-NOESY (red) spectra to highlight the NOE connectivity arising from these glycosidic linkages. Full arrows indicate definite NOE connections, while dashed arrows indicate tentatively identified ones.
**C** NMR analysis of the T-α-D-GlcA-(1 → 2)-β-D-Xyl side chain to backbone linkage, using *1*H strip plots for *1*H,*1*H-TOCSY (black) and *1*H,*1*H-NOESY (red) spectra to highlight the NOE connectivity arising from this glycosidic linkage.

**Supporting Figure** **S3** Differential activity of GT61 xylosyltransferases from *Pseudotsuga menziesii* group II (PmXYXT2) and III (PmXYXT1)
**A** PACE analysis of the *in vitro* activity of PmXYXT2 and PmXYXT1 on Arabidopsis de-acetylated xylan acceptor, which was hydrolysed after the enzymatic reaction, with xylanase GH11, glucuronidase GH115, GH51 and GH62 as indicated. pEAQ: empty vector control. S: Standard of xylo-oligosaccharides X_1_-X_6_.
**B** PACE analysis of xylan oligosaccharides from stem tissue heterologously expressing *pIRX3**::PmXYXT2* hydrolysed by xylanase GH11. Expression in WT, *gux1 gux2* and *esk1 kak8* was analysed compared to their respective genetic background plants. #1 and #2 shows the results of two independent transgenic lines.
**C** PACE analysis of xylan oligosaccharides from stem tissue heterologously expressing *pIRX3**::PmXYXT1* hydrolysed by xylanase GH11 and GH62 as indicated. Expression in WT, *gux1 gux2* and *esk1 kak8* was analysed compared to untransfected plants.
Note: Gels in B and C were run with 10% acrylamide (instead of the standard 20%), causing Y and Y′ to migrate slightly faster.

**Supporting Figure** **S4** Subcellular localisation of PmXYXT2 in *Nicotiana benthamiana* leaves.
PmXYXT2 localises to the Golgi apparatus. Transient co-expression of *35S**::PmXYXT2-GFP* (cyan) and 35S::ManI-mCherry (*magenta*) in tobacco leaves imaged by confocal fluorescence microscopy, showing co-localisation at the Golgi in the merged image (*white*). Scale bar 5 μm.

**Supporting Figure** **S5** PACE analysis of xylan oligosaccharides generated by ectopic expression of GT61 xylosyltransferases in Arabidopsis stem
**A** PACE analysis of *esk1 kak8* xylan expressing AtXYXT1/MUCI21/MUM5, OsXYXT1 and PmXYXT2 under the *IRX3* promoter. AIR from stem was hydrolysed by xylanase GH11, GH115 and arabinofuranosidase GH51 as indicated. Control: *Metasequoia glyptostroboides* wood. Asterisks indicate additional, unidentified oligosaccharides that likely represent more complex xylosylated xylan structures. S: Standard is the xylose ladder X-X_6_.
B PACE analysis of AtXYXT1 overexpression in Arabidopsis WT hydrolysed with xylanase GH30 and GH3. YXXXUX (orange arrow).

**Supporting Figure** **S6** Arabidopsis AtXYXT2 and AtXYXT3 mutants
**A** Schematic representation of Arabidopsis T-DNA insertion lines of *AtXYXT2* (top) and RT-PCR confirming the knock-out via T-DNA insertion (bottom).
**B** Schematic representation of CRISPR/Cas9 guide RNA (gRNA) target design (top), sequencing of CRISPR/Cas9 lines used in this analysis (bottom).

**Supporting Figure** **S7** Analysis of seed phenotype in *atxyxt1 atxyxt2 atxyxt3 mutants*
**A** Seed expression of *AtXYXT1*, *AtXYXT2* and *AtXYXT3* in the seed coat obtained from eFP browser (<http://bar.utoronto.ca/efp_arabidopsis/cgi-bin/efpWeb.cgi>).
**B** Mucilage phenotype in *atxyxt1*, *atxyxt2*, *atxyxt1 atxyxt2* and *atxyxt1 atxyxt2 atxyxt3*. Scale bar top panel = 200 μm, bottom panel = 500 μm. Seeds were imbibed in water, gently shaken to remove non-adherent mucilage, stained with 0.01% ruthenium red, rinsed, and imaged using a Keyence BZ-X7000 microscope.

**Supporting Figure** **S8** Analysis of macro-phenotype of *atxyxt1 atxyxt2 atxyxt3* triple mutants
**A** Expression data points of the *AtXYXT* family members, obtained from eFP browser. (<http://bar.utoronto.ca/efp_arabidopsis/cgi-bin/efpWeb.cgi>)
**B** Expression heat map of *AtXYXT* family members in developing leaves, obtained from eFP browser. (<http://bar.utoronto.ca/efp_arabidopsis/cgi-bin/efpWeb.cgi>
**C** Photographs of 8-week-old Arabidopsis plants showing WT, single mutants (*atxyxt1*, *atxyxt2*, *atxyxt3*), the double mutant (*atxyxt1 atxyxt2*) and two independent triple mutants (*atxyxt1 atxyxt2 atxyxt3-1* and *-2*). Mutant lines display visibly delayed senescence compared to WT, with leaves remaining greener for longer. The effect is subtle in single mutants, more pronounced in the double mutant and most evident in the triple mutants, indicating a cumulative impact of *AtXYXT* gene disruption on leaf ageing and senescence timing. Scale bar = 5 cm.
