# Supplementary figures and images for: GT61 β‐1,2‐xylosyltransferases define a conserved xylan modification in gymnosperm and Arabidopsis primary cell walls
Source: Plant J. 2025 Nov 2;124(3):e70545. doi: 10.1111/tpj.70545 (PMC12579940; doi:10.1111/tpj.70545)

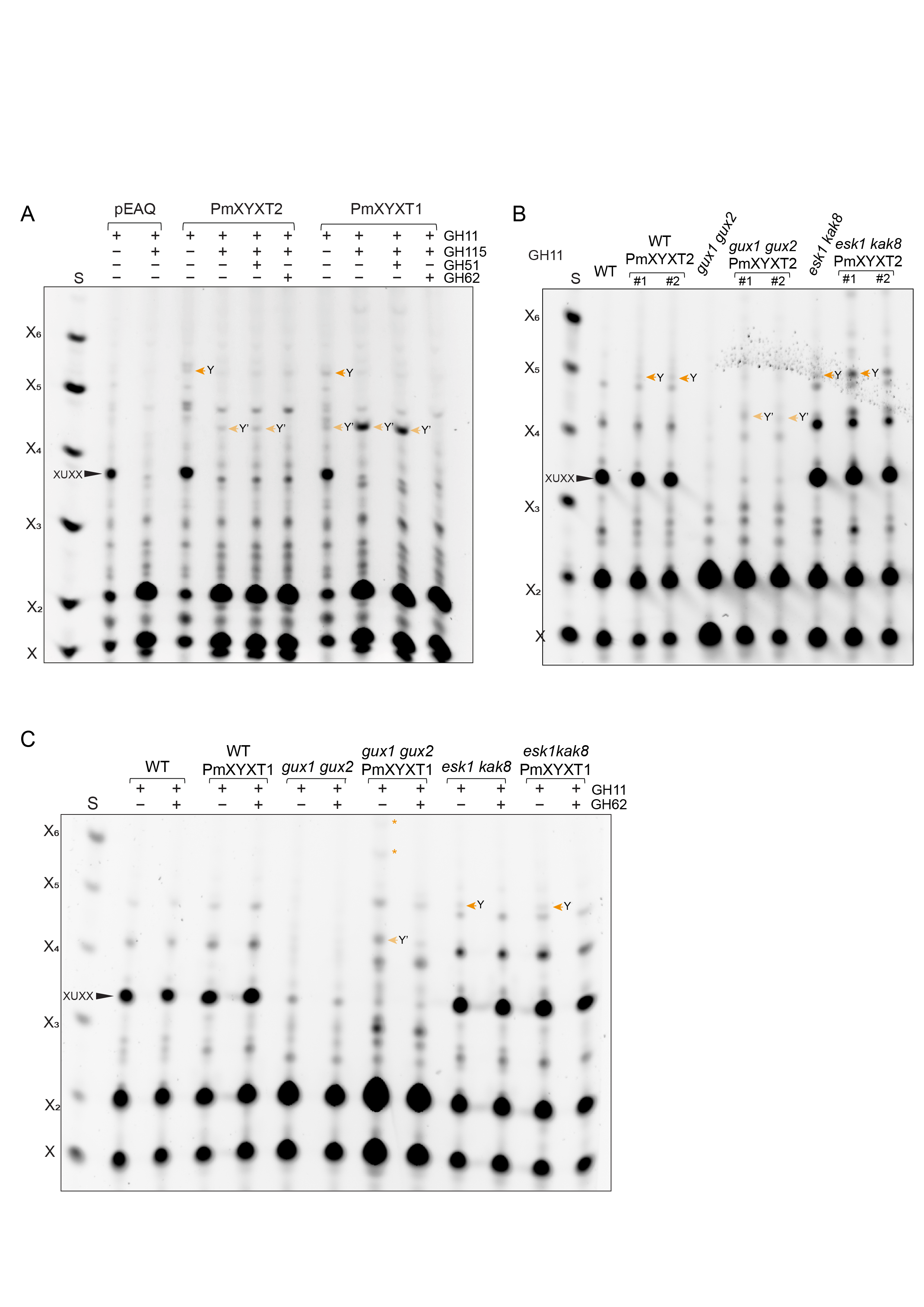

Supplement: Supplementary file 3 — Figure S1. Difference in xylan structure in needles (N) and wood (W). Figure S2. Structural analysis of Y xylo‐oligosaccharide from Metasequoia needles. Figure S3. Differential activity of GT61 xylosyltransferases from Pseudotsuga menziesii group II (PmXYXT2) and III (PmXYXT1). Figure S4. Subcellular localisation of PmXYXT2 in Nicotiana benthamiana leaves. Figure S5. PACE analysis of xylan oligosaccharides generated by ectopic expression of GT61 xylosyltransferases in Arabidopsis stem. Figure S6. Arabidopsis AtXYXT2 and AtXYXT3 mutants. Figure S7. Analysis of seed phenotype in atxyxt1 atxyxt2 atxyxt3 mutants. Figure S8. Analysis of macro‐phenotype of atxyxt1 atxyxt2 atxyxt3 triple mutants. [file TPJ-124-0-s001.zip › tpj70545-sup-0005-FigureS3.png]

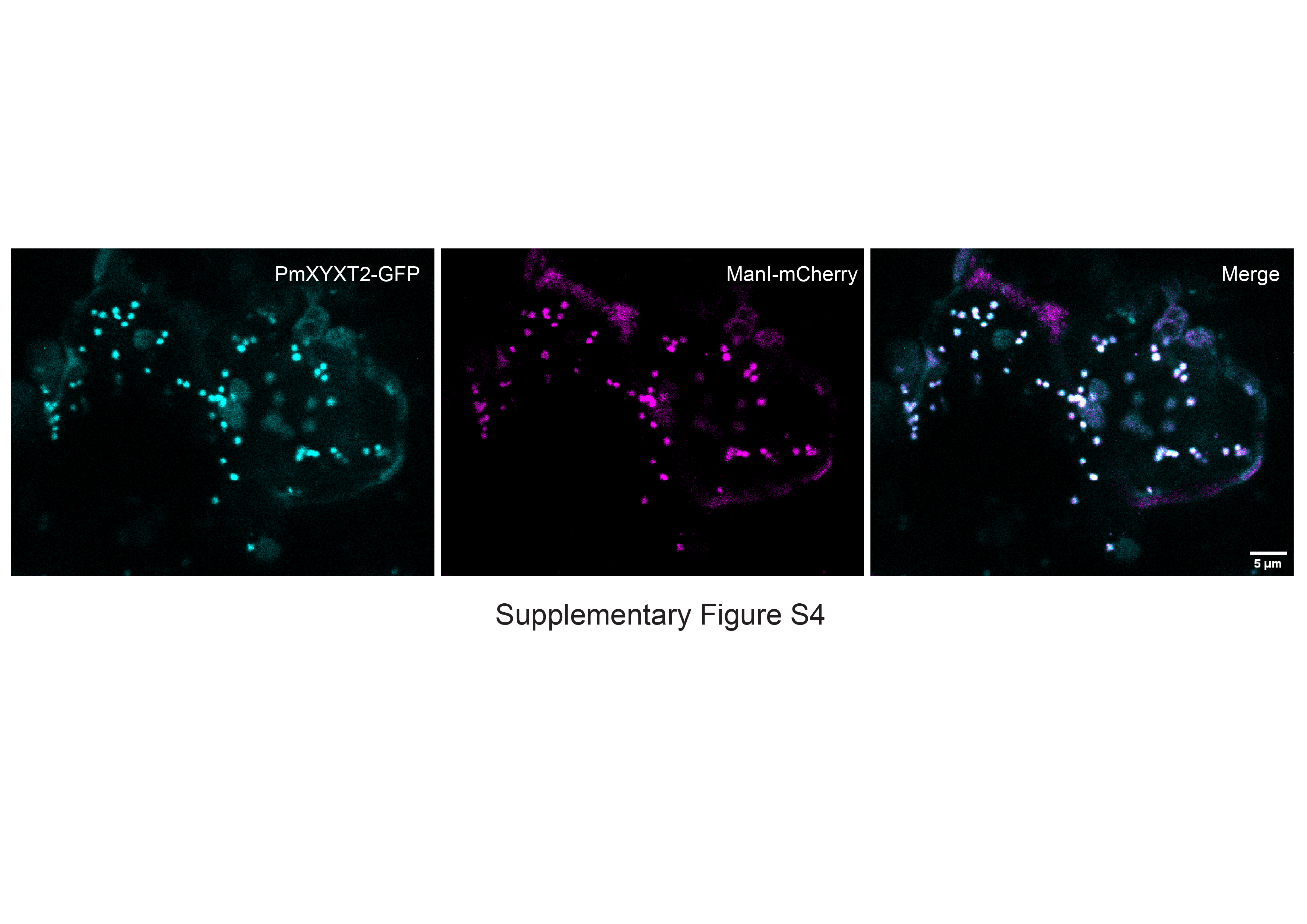

Supplement: Supplementary file 3 — Figure S1. Difference in xylan structure in needles (N) and wood (W). Figure S2. Structural analysis of Y xylo‐oligosaccharide from Metasequoia needles. Figure S3. Differential activity of GT61 xylosyltransferases from Pseudotsuga menziesii group II (PmXYXT2) and III (PmXYXT1). Figure S4. Subcellular localisation of PmXYXT2 in Nicotiana benthamiana leaves. Figure S5. PACE analysis of xylan oligosaccharides generated by ectopic expression of GT61 xylosyltransferases in Arabidopsis stem. Figure S6. Arabidopsis AtXYXT2 and AtXYXT3 mutants. Figure S7. Analysis of seed phenotype in atxyxt1 atxyxt2 atxyxt3 mutants. Figure S8. Analysis of macro‐phenotype of atxyxt1 atxyxt2 atxyxt3 triple mutants. [file TPJ-124-0-s001.zip › tpj70545-sup-0006-FigureS4.png]

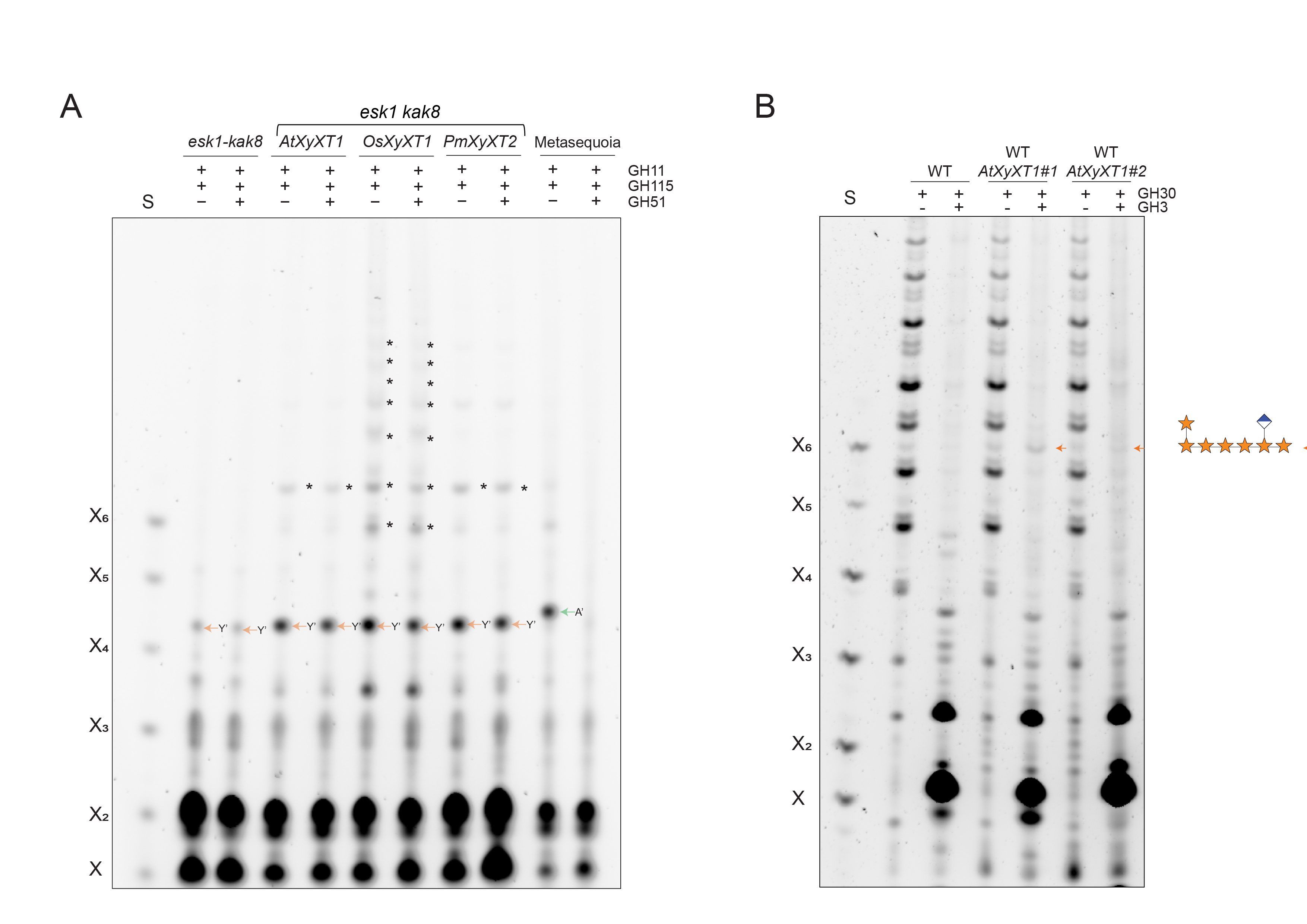

Supplement: Supplementary file 3 — Figure S1. Difference in xylan structure in needles (N) and wood (W). Figure S2. Structural analysis of Y xylo‐oligosaccharide from Metasequoia needles. Figure S3. Differential activity of GT61 xylosyltransferases from Pseudotsuga menziesii group II (PmXYXT2) and III (PmXYXT1). Figure S4. Subcellular localisation of PmXYXT2 in Nicotiana benthamiana leaves. Figure S5. PACE analysis of xylan oligosaccharides generated by ectopic expression of GT61 xylosyltransferases in Arabidopsis stem. Figure S6. Arabidopsis AtXYXT2 and AtXYXT3 mutants. Figure S7. Analysis of seed phenotype in atxyxt1 atxyxt2 atxyxt3 mutants. Figure S8. Analysis of macro‐phenotype of atxyxt1 atxyxt2 atxyxt3 triple mutants. [file TPJ-124-0-s001.zip › tpj70545-sup-0007-FigureS5.png]

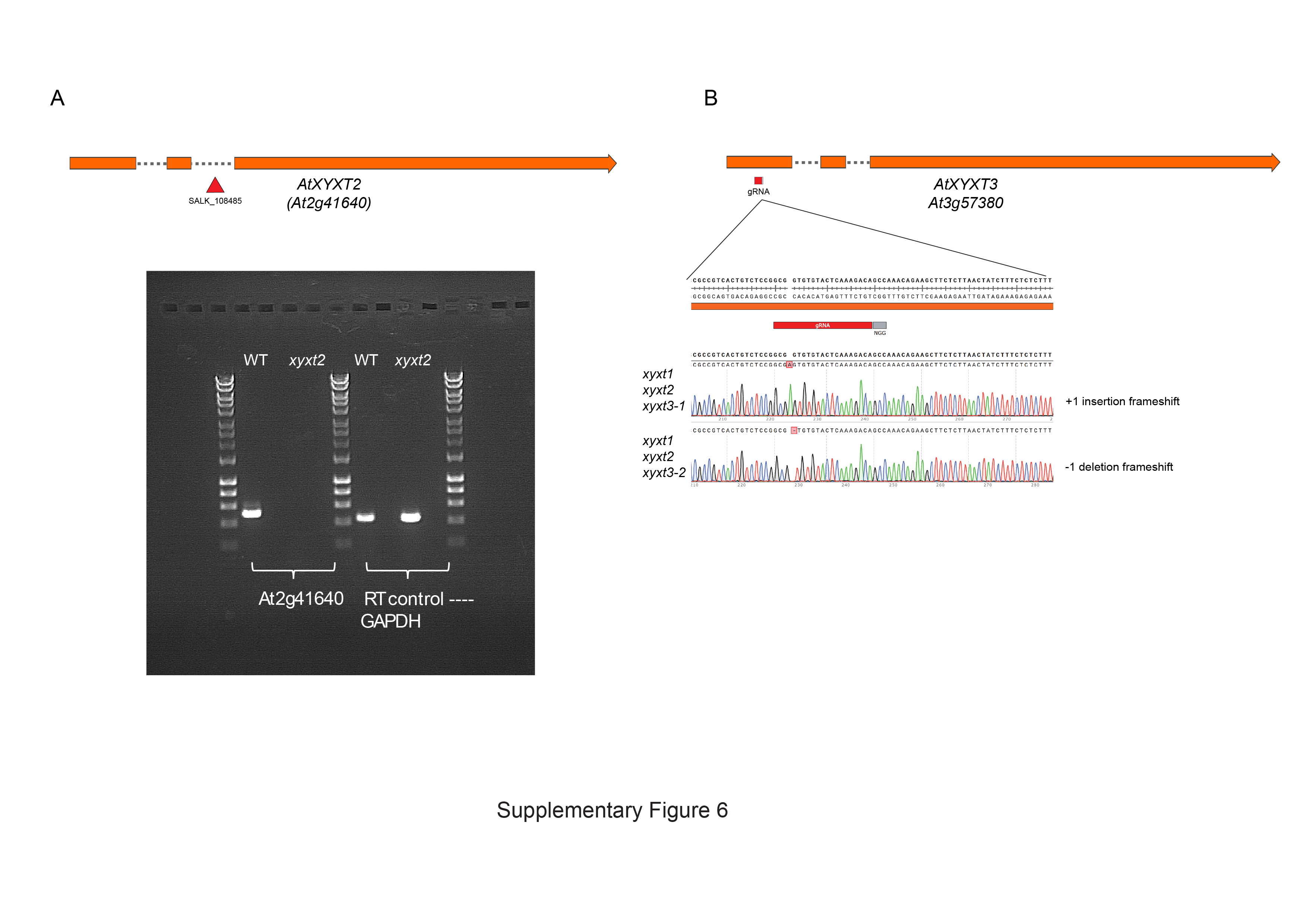

Supplement: Supplementary file 3 — Figure S1. Difference in xylan structure in needles (N) and wood (W). Figure S2. Structural analysis of Y xylo‐oligosaccharide from Metasequoia needles. Figure S3. Differential activity of GT61 xylosyltransferases from Pseudotsuga menziesii group II (PmXYXT2) and III (PmXYXT1). Figure S4. Subcellular localisation of PmXYXT2 in Nicotiana benthamiana leaves. Figure S5. PACE analysis of xylan oligosaccharides generated by ectopic expression of GT61 xylosyltransferases in Arabidopsis stem. Figure S6. Arabidopsis AtXYXT2 and AtXYXT3 mutants. Figure S7. Analysis of seed phenotype in atxyxt1 atxyxt2 atxyxt3 mutants. Figure S8. Analysis of macro‐phenotype of atxyxt1 atxyxt2 atxyxt3 triple mutants. [file TPJ-124-0-s001.zip › tpj70545-sup-0008-FigureS6.png]

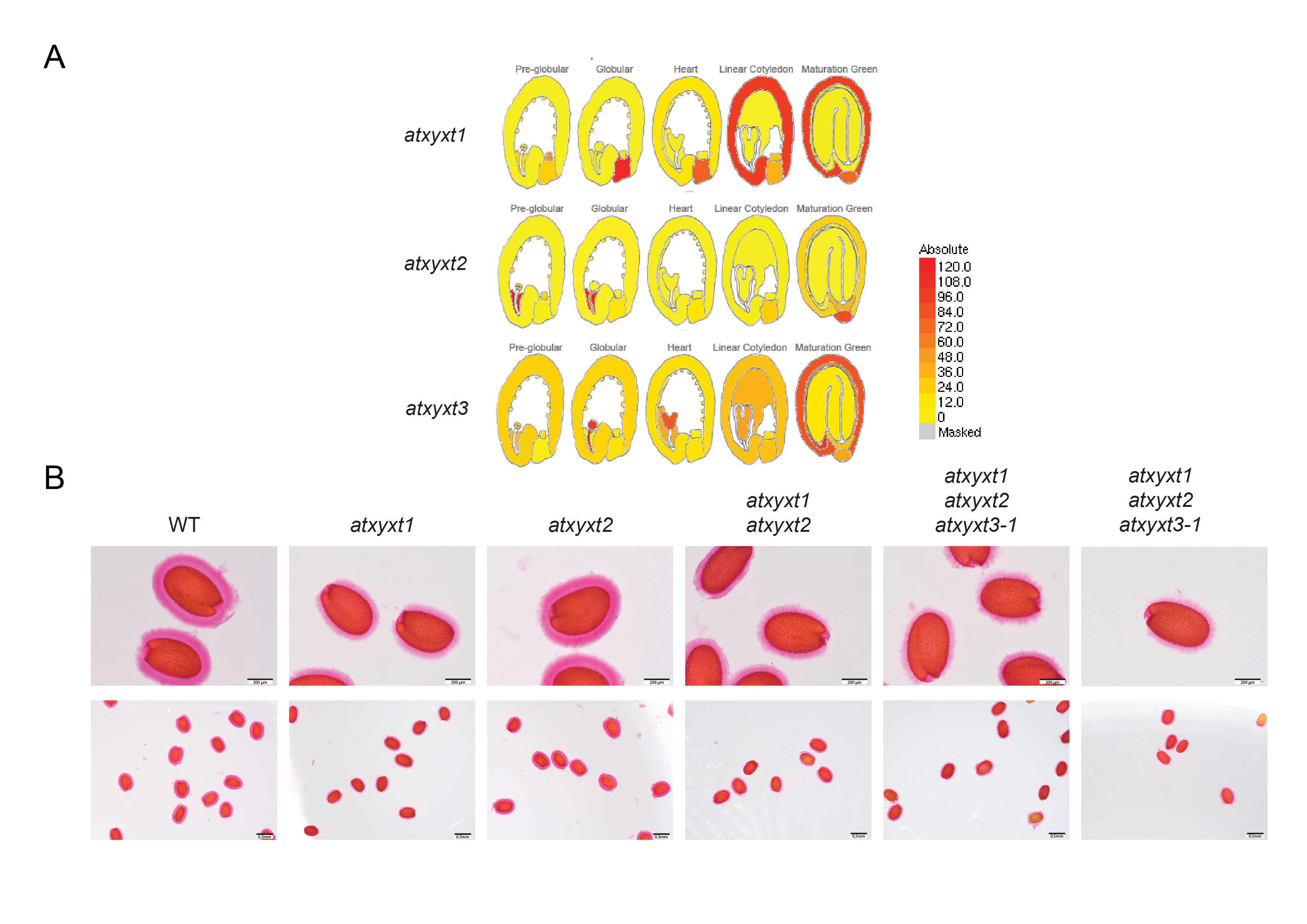

Supplement: Supplementary file 3 — Figure S1. Difference in xylan structure in needles (N) and wood (W). Figure S2. Structural analysis of Y xylo‐oligosaccharide from Metasequoia needles. Figure S3. Differential activity of GT61 xylosyltransferases from Pseudotsuga menziesii group II (PmXYXT2) and III (PmXYXT1). Figure S4. Subcellular localisation of PmXYXT2 in Nicotiana benthamiana leaves. Figure S5. PACE analysis of xylan oligosaccharides generated by ectopic expression of GT61 xylosyltransferases in Arabidopsis stem. Figure S6. Arabidopsis AtXYXT2 and AtXYXT3 mutants. Figure S7. Analysis of seed phenotype in atxyxt1 atxyxt2 atxyxt3 mutants. Figure S8. Analysis of macro‐phenotype of atxyxt1 atxyxt2 atxyxt3 triple mutants. [file TPJ-124-0-s001.zip › tpj70545-sup-0009-FigureS7.png]

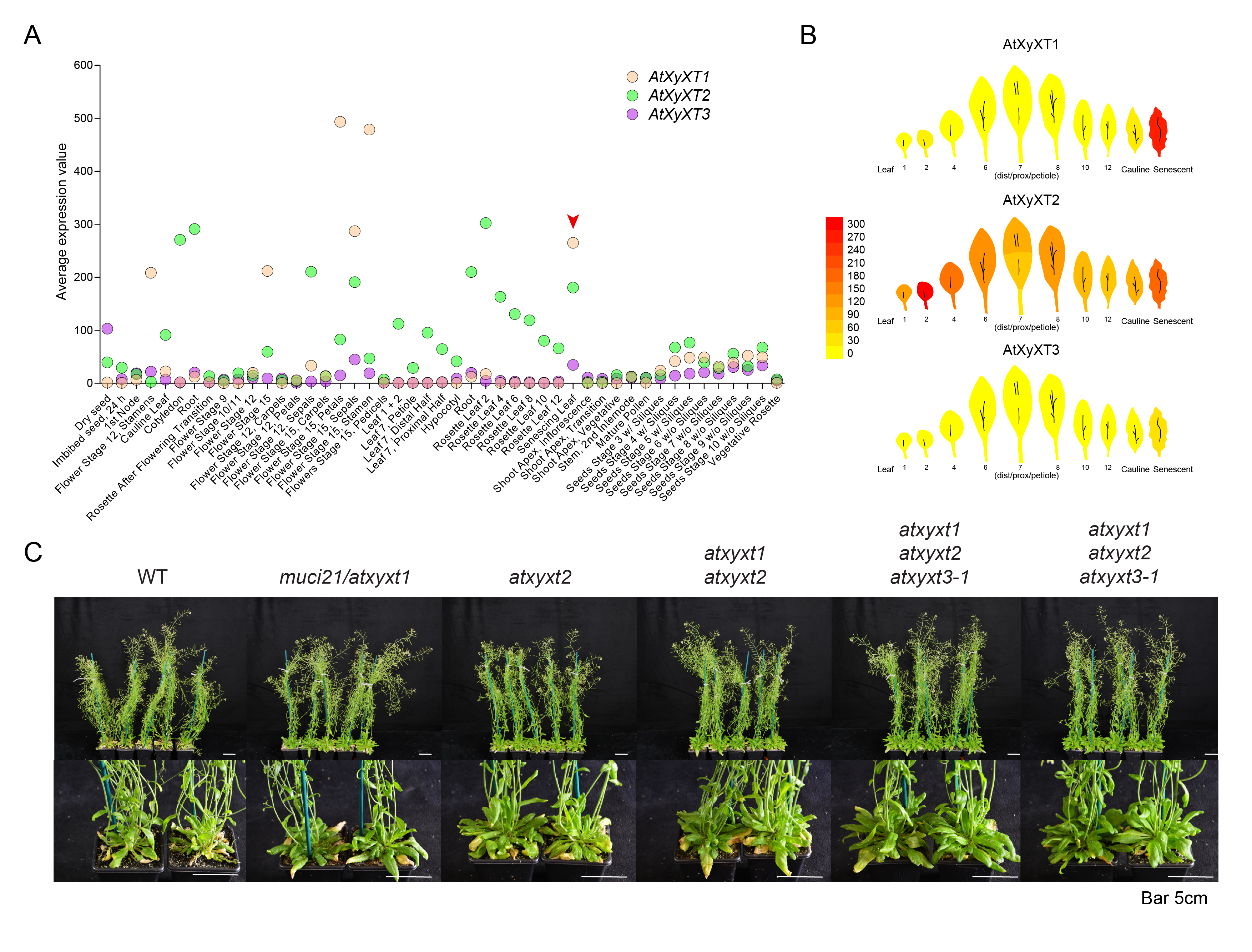

Supplement: Supplementary file 3 — Figure S1. Difference in xylan structure in needles (N) and wood (W). Figure S2. Structural analysis of Y xylo‐oligosaccharide from Metasequoia needles. Figure S3. Differential activity of GT61 xylosyltransferases from Pseudotsuga menziesii group II (PmXYXT2) and III (PmXYXT1). Figure S4. Subcellular localisation of PmXYXT2 in Nicotiana benthamiana leaves. Figure S5. PACE analysis of xylan oligosaccharides generated by ectopic expression of GT61 xylosyltransferases in Arabidopsis stem. Figure S6. Arabidopsis AtXYXT2 and AtXYXT3 mutants. Figure S7. Analysis of seed phenotype in atxyxt1 atxyxt2 atxyxt3 mutants. Figure S8. Analysis of macro‐phenotype of atxyxt1 atxyxt2 atxyxt3 triple mutants. [file TPJ-124-0-s001.zip › tpj70545-sup-0010-FigureS8.png]

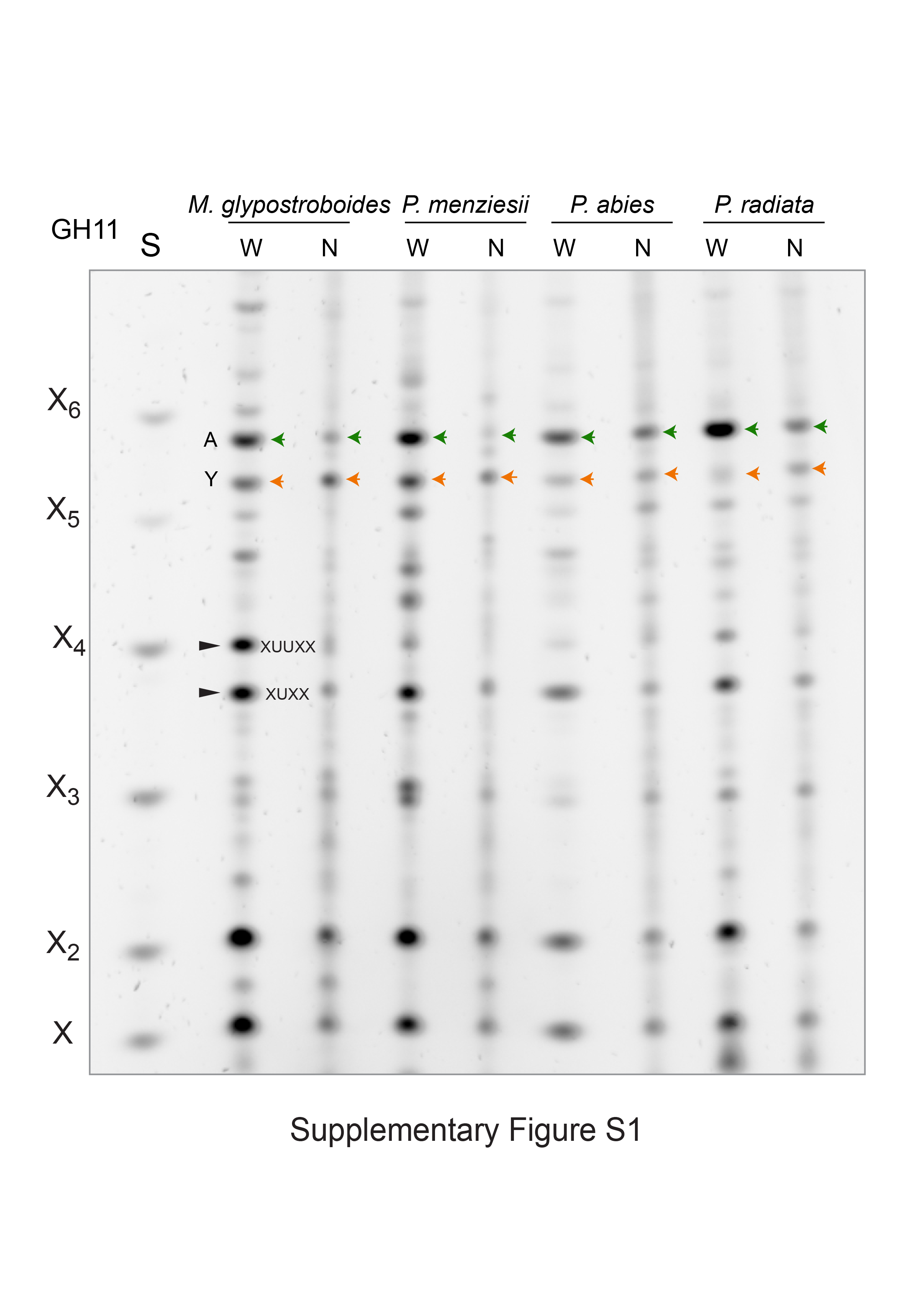

Supplement: Supplementary file 3 — Figure S1. Difference in xylan structure in needles (N) and wood (W). Figure S2. Structural analysis of Y xylo‐oligosaccharide from Metasequoia needles. Figure S3. Differential activity of GT61 xylosyltransferases from Pseudotsuga menziesii group II (PmXYXT2) and III (PmXYXT1). Figure S4. Subcellular localisation of PmXYXT2 in Nicotiana benthamiana leaves. Figure S5. PACE analysis of xylan oligosaccharides generated by ectopic expression of GT61 xylosyltransferases in Arabidopsis stem. Figure S6. Arabidopsis AtXYXT2 and AtXYXT3 mutants. Figure S7. Analysis of seed phenotype in atxyxt1 atxyxt2 atxyxt3 mutants. Figure S8. Analysis of macro‐phenotype of atxyxt1 atxyxt2 atxyxt3 triple mutants. [file TPJ-124-0-s001.zip › tpj70545-sup-0003-FigureS1.png]

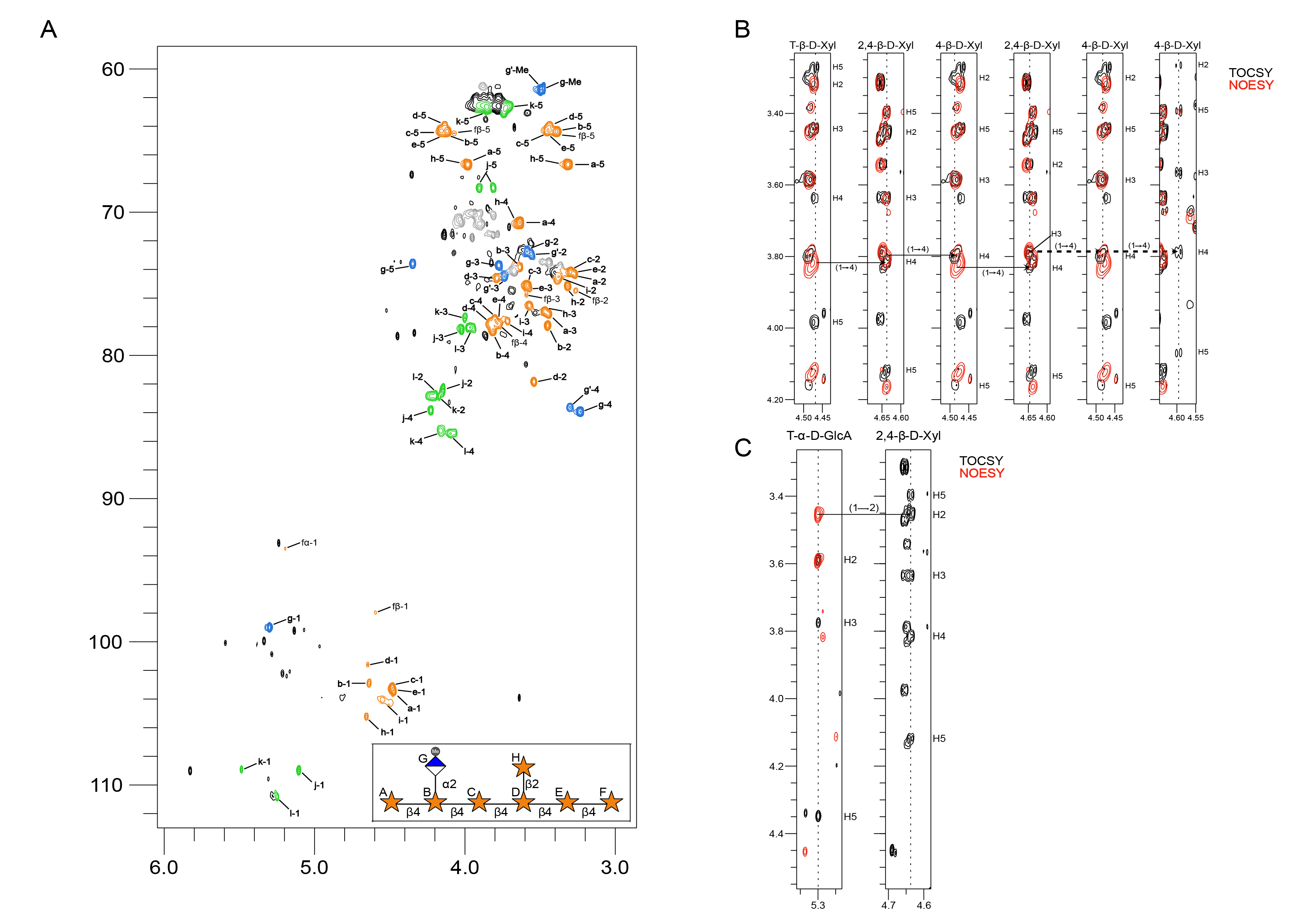

Supplement: Supplementary file 3 — Figure S1. Difference in xylan structure in needles (N) and wood (W). Figure S2. Structural analysis of Y xylo‐oligosaccharide from Metasequoia needles. Figure S3. Differential activity of GT61 xylosyltransferases from Pseudotsuga menziesii group II (PmXYXT2) and III (PmXYXT1). Figure S4. Subcellular localisation of PmXYXT2 in Nicotiana benthamiana leaves. Figure S5. PACE analysis of xylan oligosaccharides generated by ectopic expression of GT61 xylosyltransferases in Arabidopsis stem. Figure S6. Arabidopsis AtXYXT2 and AtXYXT3 mutants. Figure S7. Analysis of seed phenotype in atxyxt1 atxyxt2 atxyxt3 mutants. Figure S8. Analysis of macro‐phenotype of atxyxt1 atxyxt2 atxyxt3 triple mutants. [file TPJ-124-0-s001.zip › tpj70545-sup-0004-FigureS2.png]
